# Electronic informed consent criteria for research ethics review: a scoping review

**DOI:** 10.1186/s12910-022-00849-x

**Published:** 2022-11-21

**Authors:** Mohd Yusmiaidil Putera Mohd Yusof, Chin Hai Teo, Chirk Jenn Ng

**Affiliations:** 1 Centre of Oral & Maxillofacial Diagnostics and Medicine Studies, Faculty of Dentistry, Universiti Teknologi MARA, Sungai Buloh Campus, 47000 Sungai Buloh, Selangor Malaysia; 2 Institute of Pathology, Laboratory and Forensic Medicine (I-PPerForM), Universiti Teknologi MARA, Sungai Buloh Campus, Jalan Hospital, 47000 Sungai Buloh, Selangor Malaysia; 3 UM eHealth Unit, Faculty of Medicine, Universiti Malaya, 50603 Kuala Lumpur, Malaysia; 4 Department of Primary Care Medicine, Faculty of Medicine, Universiti Malaya, 50603 Kuala Lumpur, Malaysia; 5 SingHealth Duke-NUS Academic Medical Centre, Singapore, 169856 Singapore

**Keywords:** Electronic informed consent, Institutional review board, Research ethics committee, Autonomy

## Abstract

**Background:**

The research shows a growing trend in using an electronic platform to supplement or replace traditional paper-based informed consent processes. Instead of the traditionally written informed consent document, electronic informed consent (eConsent) may be used to assess the research subject’s comprehension of the information presented. By doing so, respect for persons as one of the research ethical principles can be upheld. Furthermore, these electronic methods may reduce potential airborne infection exposures, particularly during the pandemic, thereby adhering to the beneficence and nonmaleficence principle. This scoping review aims to identify the ethics related criteria that have been included in electronic informed consent processes and to synthesize and map these criteria to research ethics principles, in order to identify the gaps, if any, in current electronic informed consent processes.

**Methods:**

The search was performed based on internet search and three main databases: PubMed, SCOPUS and EBSCO. PRISMA Extension for Scoping Reviews (PRISMA-ScR): Checklist and Explanation guideline was used to report this work.

**Results:**

Of 34 studies that met the inclusion criteria, 242 essential original constructs were collated, and 7 concepts were derived. Digital content showed the highest percentage of collated original constructs (27%, n = 65) followed by accessibility (24%, n = 56), comprehension engagement (18%, n = 43), autonomy (14%, n = 34), confidentiality (11%, n = 25), language (5%, n = 13), and parental consent (1%, n = 2). Twenty-five new items were synthesized for eConsent criteria which may provide guidance for ethical review of research involving eConsent.

**Conclusion:**

The current study adds significant value to the corpus of knowledge in research ethics by providing ethical criteria on electronic informed consent based on evidence-based data. The new synthesized items in the criteria can be readily used as an initial guide by the IRB/REC members during a review process on electronic informed consent and useful to the future preparation of a checklist.

**Supplementary Information:**

The online version contains supplementary material available at 10.1186/s12910-022-00849-x.

## Background

Informed consent acts as a safeguard for the potential research participants to be informed about the nature of the research trials they are requested to participate [1]. Potential research participants need to be informed about the research procedures; the potential risks, possible benefits; autonomy or right to decline to participate or withdraw without being penalized following agreement to participate; assurance on confidentiality issues; reimbursement, compensation and incentives for participating; and the contact information of the researchers should the participant have any concerns about the study [2]. This information is generally disclosed through a participant information sheet coupled with the participant's written informed consent document before participating in the study.

In several investigations to assess comprehension and recall of informed consent and related legal informational documents, participants were reported to not reading or only skimming the material, and recall findings support these reports [3, 4]. Entrusting the researcher, not having time to read the document, and having had the document verbally explained are among the common motives for not reading these documents [5]. In addition, participants’ comprehension of and recall for the information is affected by readability and vocabulary of the document based on their age, education, and cognitive and mental status even when they carefully read informed consent [6]. Not reading or not understanding (or both) informed consent documents may lead to severe consequences. Stanley and Guido found that participants in some medical research studies did not even realize they were participating in research [7].

The Belmont Report categorizes three basic ethical principles for conducting research that involve human participants and expounds guidelines to assure these principles are to be abided throughout the research process [8]. The basic ethical principles include respect for persons, beneficence, and justice to the human research participants. In general, the use of electronic informed consent has been associated with increased comprehension among the research participants and hence, enhancing the beneficence [9, 10]. The principles highlighted in this report are pivotal and will be used as principal guidance in this study.

With the growing use of digital health tools, electronic informed consent (eConsent) has become a crucial element in health research and standard clinical care, especially during and post- Covid-19 pandemic [11, 12]. eConsent enables sites to continue to consent and re-consent patients in clinical trials during the time of self-isolation [13]. Not only does it allow research participants to consent in the comfort of their home, but it also provides quantitative and qualitative data that is not readily available through traditional paper consent forms [11]. According to Chen et al., 2020, there was no unified approach or guideline for replacing paper-based consent with eConsent.

Despite an increasing number of studies utilizing electronic informed consent, the reviewing process of this type of informed consent across the ethical committee may be inconsistent and incomprehensive especially when the committee is facing different electronic media such as graphics, audio, video, podcasts, and websites [14, 15]. As the electronic informed consent is different from paper-based informed consent, problems may arise that could impair the participant's autonomy to make decisions. The objectives of this study are twofold: (1) to identify the criteria in ethical conduct of electronic informed consent taking; and (2) to synthesize and map these criteria to research ethics principles, in order to identify the gaps, if any, in current electronic informed consent processes.

## Methodology

The main methodological approach for this research is a scoping review. Generally, scoping reviews aim to map literature on a particular topic and explore the underpinnings of a research area, as well as identify and clarify the key concepts, theories, sources of evidence and gaps in the research [16].

**Fig. 1:**
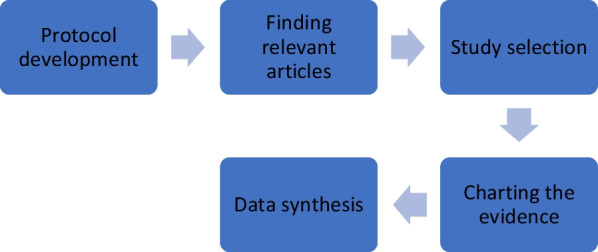
Scoping review flowchart

In this first phase of the study, scoping review was performed according to guidelines set forth by the Joanna Briggs Institute [17] and the Preferred Reporting Items for Systematic Review and Meta-Analyses (PRISMA) [18] for the conduct and reporting of scoping review, respectively. Methodological framework starting from identifying research questions, identifying relevant studies, study selection, charting the data, collating, summarizing, and reporting of the results was performed based on the flowchart for scoping review process as depicted in Fig. 1 [16].

Inclusion criteria for the selected articles within the current scoping review included publication time period from 1998 to 2021, English language and focusing on eConsent within the scope of health research/trials. The reason for the chosen time frame was based on the preliminary search for eConsent trend on PubMed. Studies focusing on eConsent within the scope of clinical practice and local regulations, or law were excluded from the review. This scoping review's research question is formulated based on Population, Concept, and Context (PCC) framework; Population: Any population, Concept: Electronic informed consent and Context: IRB/IEC review and oversight.

### Identification of relevant studies

A comprehensive search for relevant articles was performed using three databases that includes PubMed, SCOPUS and EBSCO. Articles from commentaries, opinion pieces, blogs and online materials were also included within this scoping review. Also, at the outset of the study, local experts from the research ethics committee were consulted to formulate a preliminary proposition on the review of the electronic informed consent within their practice. The selection of the local experts was based on their experience in the research ethics committee and availability. In addition, reference mining was performed following a full text article collection. Boolean logic using ‘OR’, ‘AND’, and ‘NOT’ and MeSH terms was utilized to enhance the search (Additional file 1: Appendix I for search strategy). A single reviewer (MYPMY) gathered data from all fully eligible studies and organized it in a data extraction excel form according to the predetermined variable headings. Another reviewer (TCH) went over the data obtained separately to ensure consistency. Any conflicts that arose were handled by NCJ as the third reviewer.

### Study selection

Under study selection, title and abstract screening, followed by full-text screening using pre-defined inclusion and exclusion criteria, was performed. All related duplicates were removed, and reference was managed using EndNote^®^ software.

### Charting the evidence

The evidence’s charting was gathered using a pre-defined, standardized charting form (Additional file 2: Appendix II) using Windows’ Microsoft Excel 2019 16.0.6742.2048. The studies were charted according to geographic area, article type, study design and population. These were reported using descriptive statistics. 

### Data synthesis

The constructs in this review are defined as the domains that are used to inform the ethical conduct and review of electronic informed consent. The ethical constructs were identified by screening and transcribing the texts of the primary articles verbatim. Prior to constructs synthesis, all items were labelled according to specific item number obtained from each primary study/resource. For constructs synthesis, all of the extracted verbatim constructs were first sorted according to common themes. The themes were then named to form the concepts for the conduct of electronic informed consent. The constructs within each concept were also synthesised through a process of rewording and merging to remove duplicative constructs. The synthesis was performed by a single review author (MYPMY) and consensus was obtained via discussions with the other two review authors (TCH and NCJ). The final framework was presented with the newly synthesized constructs under each concept.

## Results

**Fig. 2:**
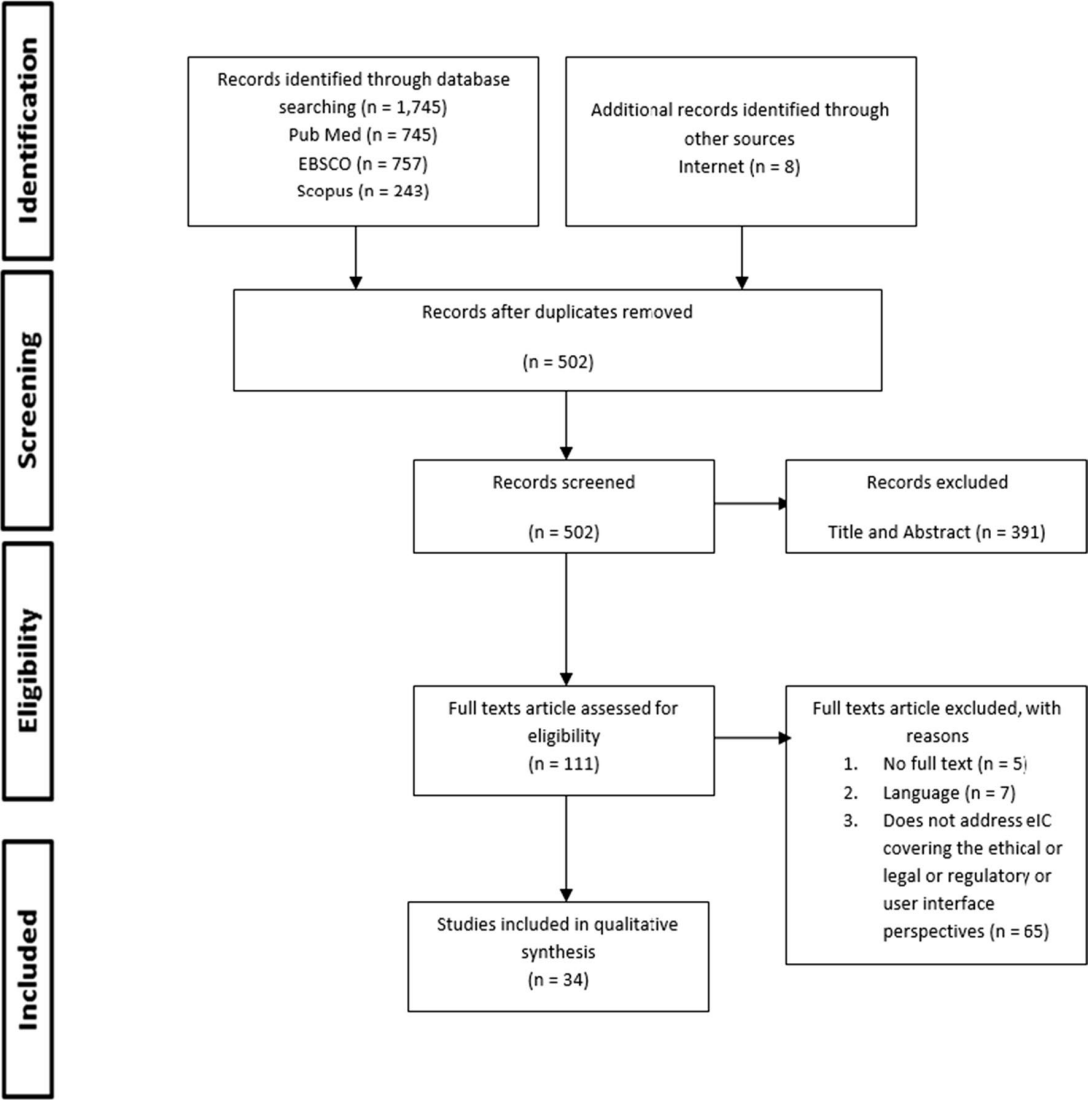
Selection process of the studies included based on PRISMA-ScR Flow Diagram

This scoping review identified a total of 1745 potentially eligible articles that were published between January 1998 to April 2021 (Fig. 2). The results were generated across three databases, namely PubMed (n = 719), SCOPUS (n = 71), and EBSCO (n = 1,087). Additional records were identified through other sources such as internet search (n = 8), and reference mining (n = 3). Out of these, 1243 articles were identified and removed due to duplication. The titles of the remaining 502 articles were screened. Following the next phase of the screening process, 391 articles were further eliminated, after title screening (n = 328) and abstract screening (n = 63). The full texts of a total of 111 papers were further reviewed and compared to fit the inclusion and exclusion criteria model.

After the full text screening process is completed, a total of 77 articles were excluded with the reasons as listed: no full text (n = 5), language other than English (n = 7), does not address electronic informed consent covering the ethical or legal or regulatory or user interface perspectives (n = 65). The search strategy was done following the PRISMA Extension for Scoping Reviews (PRISMA-ScR): Checklist and Explanation guideline [17], as described in the methodology, and as presented in Fig. 2. A total of 34 articles that fitted the inclusion criteria were included in the qualitative synthesis.

Characteristics of the selected articles and the online materials were tabulated in Table 1. The majority of the articles originated from North America (65%, n = 22) followed by Europe (32%, n = 11) and Asia (3%, n = 1). Of these, twelve articles used randomised controlled trials while thirteen articles adopted methodical/framework analysis for their study design with percentage of 35% and 38%, respectively. Interviews/surveys and scoping/systematic reviews were among the study designs performed in primary studies. Overall, the area of the studies displayed a wide extent of topics ranging from electronic health records (36%, n = 13) to cybersecurity (3%, n = 1). Most documents were from academic researchers.

**Table 1:** Characteristics of articles and online materials meeting the inclusion criteria

Domain of interest	N = 34 (%)
*Geographic area*	
North America, n (%)	22 (65)
Europe	11 (32)
Asia	1 (3)
*Article type*	
Original research	18 (50)
Perspective	12 (39)
Review	4 (11)
*Study design*	
Randomised controlled trial	12 (35)
Methodical/framework analysis	13 (38)
Interviews/surveys	6 (18)
Scoping/systematic review	3 (9)
*Area of study*	
Electronic health records	13 (36)
Surgery	4 (11)
HPV and HIV research	3 (8)
Stakeholder groups	3 (8)
App development	6 (22)
Patients underrepresented in research	2 (6)
Cybersecurity	1 (3)
Regulations and guidelines	2 (6)

Of the 34 eligible articles, 242 essential original constructs were collated. All original constructs were tabulated according to the authors and their specific title (Additional file 3: Appendix III). Original constructs within the context of this study are defined as the underlying ethical themes in conducting and reviewing the electronic informed consent. 32 articles were academic resources while 2 articles were internet-based (United States Food and Drug Administration and REDCap).

Of the 242 original constructs, four were deleted (Items 34.5, 34.6, 34.7, 34.8). The four items were deleted due to specific requirements from local regulations and laws. All four deleted items were from the same study [19]. The remaining 238 items were modified through a process of rewording and merging. Subsequently, seven concepts for the conduct of electronic informed consent were established: accessibility, autonomy, engagement for comprehension, confidentiality, digital content, language, and parental consent. In general, 65 items were categorized under the concepts of digital content followed with accessibility (n = 55), engagement for comprehension (n = 42), autonomy (n = 33), confidentiality (n = 24), language (n = 12), and parental consent (n = 2). The summarized descriptions were further synthesized resulting in the development of 25 newly synthesized constructs (Table 2).

**Table 2:** New synthesized constructs for electronic informed consent

Engagement for comprehension	Digital content	Accessibility	Parental consent	Language	Confidentiality	Autonomy
1. Undertaking the consent process before the day of intervention	1. Establish digital technology for participants to track the use of their biological sample over time	1. Identify the demographics and needs of the participants	1. Give opportunity to gather information from parents/LAR	1. Simplify the language	1. Use encryption for data in transit	1. Participant is always able to control and restrict the access to the shared record
2. Support social annotation whereby participants can see each other comments for discussions	2. Use validated electronic signature	2. Establish tracking mechanism for separate consents		2. Use multiple language	2. Provide secured and proper authentication	2. Use only explicit consent
3. Provide interactive personnel to optimize comprehension and trust	3. Establish digital storing biometric information on cloud storage	3. Integrate research consent data with electronic health record			3. Needs to be legally binding	3. Participant is always able to control their own pace during consent process
4. Fulfil general requirements and elements of informed consent	4. Support interactive digital components such as online quiz, graphical media and audio-visual aid	4. Participants receive a copy of the completed consent form			4. Provide options to discuss in private	4. Allow participant to amend their consent
	5. Support information technology infrastructure	5. Allow IRB oversight and amendment				

**Fig. 3:**
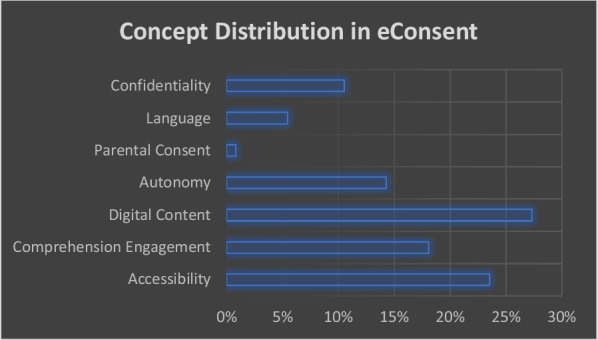
Concept distribution in electronic informed consent

Digital content showed the highest percentage of collated original constructs (27%, 65/238) followed with comprehension engagement (18%, 43/238) in concept distribution (Fig. 3). Autonomy and confidentiality exhibited 14% (34/238) and 11% (25/238), respectively. The least percentage for electronic informed consent constructs described in literature were language (5%, 13/238) and parental consent (1%, 2/238).

## Discussion

Generally, the electronic informed consent has fulfilled the general elements of informed consent. However, based on our understanding of the literature, it appears that the criteria are different from traditional pen-and-paper informed consent. As more healthcare research moves to an electronic platform, it is more important than ever to assess the criteria for conducting electronic informed consent taking processes.

The primary papers and internet materials identified in the search showed a skewed tendency to North America. Considering the broad applicability of electronic informed consent following the COVID-19 pandemic, it is postulated that more research will be performed in this topic. Nevertheless, it is also interesting to note that since the current scoping review is focusing on electronic informed consent within the context of healthcare research ethics, it may partly explain the demographic skewness. Significant number of papers were omitted during the earlier phase of the review search that were primarily performed under the contexts of clinical practice and clinical ethics.

Four original items were deleted due to their specific descriptions being based on local regulations. As this study was undertaken with the assumption that general requirements for informed consent must be free from the influences of local regulations and laws, these items were removed. Code of Federal Regulations (CFR) 11 (Electronic Records; Electronic Signatures) was the specific local regulations for all these four items. As this regulation only applies to the USA, the justification for removal is met. It is interesting to note that all these items stemmed from a single study [19, 20].

The primary reviewer and research team members met frequently to improve the reliability and quality of the study selection in this scoping review. Differences and disagreements were laid out on the table, and a consensus was established. It is worth noting that the present scoping study did not use the same quality rating tools as the systematic review, such as CASP or Cochrane RoB 2 [17]. Therefore, although the use of such a tool is not generally a practice in conducting and reporting scoping review, the lack of it may reflect a limitation in this study. In addition, seven key concepts that hold twenty-five new synthesized items for the identified criteria of electronic informed consent have been framed within this study.

### Accessibility

Accessibility is the top two important concepts formulated for the conduct of informed consent in this study. Chen and his team through REDCap-based workflow for electronic informed consent had outlined most of their frameworks of electronic informed consent on accessibility [19]. In fact, with thirty-six original items on their study alone, they have become the group’s largest contributor. However, as their work on REDCap was based on specific federal guidance in the United States of America (USA), some of the items were omitted. In addition, another Switzerland-based study using Fast Healthcare Interoperability Resources (FHIR) on the conduct of electronic informed consent taking suggested implementing a custom label reader to retrieve participant data needed for the consent document, such as name, date of birth and participant special identification number [21]. It is important to note that both REDCap and FHIR works were developed according to local regulatory settings and the applicability of such systems may require validations prior to broad implementations.

As far as the accessibility goes, the involvement of IRB/REC is important in reviewing the electronic informed conduct. The oversight and review of the conventional informed consent is already well established in almost all local IRB/REC settings [22]. Therefore, the access to institutional review board purview was established as part of the twenty-five preliminary checklist items in this study.

### Autonomy

The mechanism to allow participants to amend their consent and access the shared record and issues on timing and how participants will be able to control their pace during the process of electronic informed consent were considered in upholding the autonomy to research participants. The use of explicit consent as opposed to implicit and opt-out consent were also key issues in this preliminary checklist item. Consent can be changed in a variety of ways, from protocol updates and changes to withdrawal to reflect the autonomy.

A dynamic consent model was presented mainly in biobank studies, which allows for personalization and flexibility, such as allowing a biobank member to update their broad consent based on new research activity [23]. Dynamic electronic informed consent is defined as using technology to maintain ongoing engagement with participants in order to retain their consent choices [24]. Interestingly, several studies have stated that participants may only desire to consent to providing restricted data to for-profit businesses [25–27]. There are a variety of technical techniques to tracking this data electronically, ranging from blockchain [28] to other models [21]. Two studies described reputable third-party entities that would keep the data safe and control who had access to it [28, 29].

The processes for allowing consent revisions at various levels of attainment by research participants need to be reviewed by the IRB/REC. Therefore, we recommend that researchers either provide a screen capture of the electronic informed consent procedures to describe the mechanism in place or utilise the system that has already been implemented by institutions.

### Engagement for comprehension

Interactions and communications between researchers and participants are one of the most important factors for measuring engagement for comprehension. This in turn has the potential to foster the trust among the study participants. According to Chen et al. 2020, regardless of the form of consent, numerous studies have advocated that in-person interactions or other forms of communications with researchers remain a part of the electronic informed consent process, to ensure participants' understanding of consent information and to foster trust, especially for more complex and risky studies. Interactive personnel to optimize comprehension and trust was an essential part of developing the checklist. Large number of items were collated based on this factor. Amongst other original items/construct to depict this issue includes:

“Questions should be answered via in person discussions or combination of electronic messaging, telephone calls, video conferencing, or live chat with remotely located investigator or study personnel” [19].

“Interactive, multiple-choice questions were inserted at relevant points in the slideshow to emphasize critical concepts” [30].

“In-person interactions or other forms of communications with researchers remain a part of the eConsent process, to ensure participants’ understanding of consent information and to foster trust, particularly for more complex and riskier studies” [31].

In Item 14.3 to Item 15.8, social annotation was highlighted, and the original items were merged as ‘Support social annotation whereby participants can see each other’s comments for discussions.’ The importance of comprehension and understanding was significantly considered whereby the discussion on undertaking the consent process prior to the day of intervention was merely noted.

Some studies revealed that when electronic informed consent included interactive components, quizzes, personalized material, graphical media, and annotations, participants (including minors) had a greater knowledge of the information presented to them [30, 32–35]. In one scenario, participants with and without mental illness have the same degree of understanding, but in participants with schizophrenia, a web-aided multimedia consent may assist understanding and deliver better satisfaction [36].

In a traditional pen-and-paper informed consent, engagement for comprehension is usually not a major issue because the researcher would be present to explain everything to the research participants. However, in electronic informed consent, as most explanations will be provided by a third party, such as an animated character or avatar, IRB/REC may require that this factor be considered, as well as methods to prevent communication breakdown between research participants and researchers.

### Confidentiality

Hall and his team suggested a deduplication protocol to remove duplicate or artificial survey attempts. Their study also highlighted the potential fraudulent responses from artificial hacking or bot programs possibly aimed at getting the monetary incentive and the importance to establish indicators for that [37]. Following that, initiatives have been set up to put measures in place including Completely Automated Public Turing test to tell Computers and Humans Apart (CAPTCHA) codes and verification of email addresses submitted for incentives [37–39]. In a systematic review on the implementation of electronic informed consent in healthcare research and stakeholder’s perspectives, most participants in the study emphasized the necessity of trusting the legitimacy of electronic informed consent when sharing health information and agreeing to participate in the study [40]. Researchers believe that a secure platform will facilitate the sharing of files, which is a vital aspect in biomedical research. Individual user identities and passwords were required to access the electronic informed consent platform that Chhin and his colleagues built [41]. Furthermore, researchers stressed the importance of providing sufficient privacy information to potential research participants [42].

Confidentiality measures must be implemented in order to acquire the trust of study participants. This aspect requires regular review and control by the IRB/REC. Although researchers are expected to take proactive measures to address this problem, and ultimately performed by the data protection personnel at the institution, the IRB/REC must assure that the mechanisms to check for data protection process such as updated anti-virus and anti-malware software, as well as the use of e-consent software/platforms with embedded end-to-end encryption protection. The ethical application document must also include strategies for improved password security when using online platforms, as well as data storage processes that are transparent (particularly if they are asked to complete e-consent on portable devices, which are not their own). Using iconography (such as a padlock image) to indicate data security safeguards might also be reassuring to worried participants.

### Digital content

Digital content is another key to the implementation of electronic informed consent. Based on the several models for electronic informed consent, the use of an avatar to substitute human personnel is a plus feature for the electronic platform [43]. This feature may engage young and adolescent participants in the most significant way and therefore, it is envisaged to be highly interactive. However, the technology may require expertise and thus, may incur some costs.

The use of animations and avatars have been proposed by several studies [43–45]. To establish confidence, the avatar should appear as medical staff with lab coats and stethoscopes [43]. However, most studies agreed that avatars should not replace interactions with study staff [43, 46].

Electronic signatures for consent in the USA must comply with 21 CFR 11(c), a federal requirement that states that an electronic signature must be unique to one person and that organizations must verify that person’s identification. Researchers in Europe are subjected to comparable requirements [47]. Some IRBs were unsure whether electronic signatures were valid, which regulations would apply, and how the electronic signatures would be preserved, according to a study [48]. Therefore, in our opinion, the research teams will need to be requested to confirm whether an electronic informed consent method will be employed, and if so, whether the technology is capable of providing a verified signature while also meeting all legal criteria. A description of the recruitment and consenting approach that is consistent with how the electronic informed consent is provided will also be required. In addition, the IRB/REC will need to assess how the electronic signature is created, whether the signature can be proven to be valid, and how the research team plans to give a version of the permission form to the potential subject for review and retention when they review the study.

Secure authentication and the use of digital signatures were the inevitable component of electronic informed consent. According to Chen et al. 2020, the use of USign, a signature verification method that can integrate with existing electronic informed consent systems and provide a new authentication token should be seriously considered [31]. Electronic signature, however, is also discussed within digital consent concept as its use was separately conferred according to its contexts.

### Language

When it comes to language, most original items were in accord with each other on how the language should be presented in electronic informed consent [49–51]. Appropriateness, simplicity and concise are the adjectives used to simplify the language. It is interesting to note that although this requirement is included as one of the elements in general informed consent, the electronic informed consent has more flexibility in terms of freedom to add more language of interest. As all options are within click in electronic informed consent, the potential participants may proceed with their language of choice and thus, the principles of autonomy and comprehension will be upheld.

Because clear and simple wording is a basic requirement for informed consent, the IRB/REC may need to confirm a different version of consent based on language relevant to the study participants’ inclusion criteria in some cases.

### Parental consent

Two original items highlighted that there must be sufficient provisions for soliciting the assent of children [19, 52]. In other words, for research involving children, ample time and resources should be given to both the children and their parents to understand and reach joint consensus. Perhaps, it is an overstatement to say that children have equal portions of consensus in joint consensus. According to Hein, minors should adopt a dual consent approach from the age of 12 until they are given independent consent rights (child and parent) [53]. Based on this approach, even if a child's decision-making competence in the medical choice at hand is established, a dual consent approach will take into account developmental elements of children as well as the unique qualities of the parent–child dyad. The parental role is necessary to provide further safety by setting the stage for the child's competent decision-making and facilitating his or her long-term autonomy.

In this regard, the IRB/REC approval requires the study to verify that the study protocol is in tandem with local regulations pertaining to children. Therefore, the study team's compliance with local regulations on children should be described in the IRB/REC application. For instance, the research team could initially acquire parental consent over the phone.

Based on the research question formulated earlier in this review, using the context of research ethics review and oversight, the number of primary papers focusing on this context was limited. Therefore, it was decided to include more general research ethics criteria on electronic informed consent to identify the constructs. The ethical criteria developed in this study, however, still requires validation from the expert consensus through focus group discussion. Hence, our future work will be concentrated on developing eConsent checklists based on the criteria in this study by recruiting the expert participants amongst the IRB/REC members for validation. It is interesting to note that since the developed criteria is generic in nature, validation from the healthcare researchers may also be vitally important. This study recommends a two-pronged approach for the expert validation to work on in future with (i) IRB/REC members and (ii) healthcare researchers.

## Conclusion

The use of electronic informed consent should not be used to replace human connection; rather, technology can be used to supplement it. Participants should still be able to ask researchers questions, and they may also be able to ask each other questions through social annotations. Lack of access to and knowledge with the technology, on the other hand, can pose additional barriers to consent. As technology improves, the number of participants may grow as they are no longer restricted by their geographic closeness to the research facility. However, until that time comes, participation may skew toward younger, more affluent people who already have access.

The current study adds significant value to the corpus of knowledge in research ethics by providing criteria to conduct and review the electronic informed consent for both researchers and IRB/REC, respectively, based on evidence-based data. The new synthesized criteria can be readily used as an initial guide by the IRB/REC members during a review process on electronic informed consent.

As a result, the practice of delivering electronic informed consent will have major ramifications in terms of the advantages being more participant centric. The autonomy and beneficence to research participants will be optimized through their understanding and the use of technology to minimize infection encounters during the pandemic, respectively. As more research adapts to the use of electronic media and makes the practice of electronic informed consent inevitable in the pandemic of COVID-19, it is envisaged that there will be a great deal of research protocols pertaining to electronic forms coming to the IRB/REC. The current electronic informed consent criteria is, therefore, can be considered as a pre-emptive measure to help the IRB/REC in managing the review process.

## Supplementary Information

**Additional file 1.** Standardized Charting Form.

**Additional file 2.** Search strategy for the databases.

**Additional file 3.** Data Collection Table.

## Data Availability

The datasets generated and analyzed during the current study are not publicly available due to the security of data but are available from the corresponding author on reasonable request.

## Abbreviations

eConsent: Electronic informed consent
IRB/REC: Institutional review board/research ethics committee
MeSH: Medical subject headings
PRISMA: Preferred reporting items for systematic review and meta-analyses
REDCap: Research electronic data capture
FHIR: Fast healthcare interoperability resources
CAPTCHA: Completely automated public turing test to tell computers and humans apart
STRIDE: Strengthening translational research in diverse enrolment
