# Supplementary figures and images for: Electronic informed consent criteria for research ethics review: a scoping review
Source: BMC Med Ethics. 2022 Nov 21;23:117. doi: 10.1186/s12910-022-00849-x (PMC9682656; doi:10.1186/s12910-022-00849-x)

# Appendix I

Standardized Charting Form

**
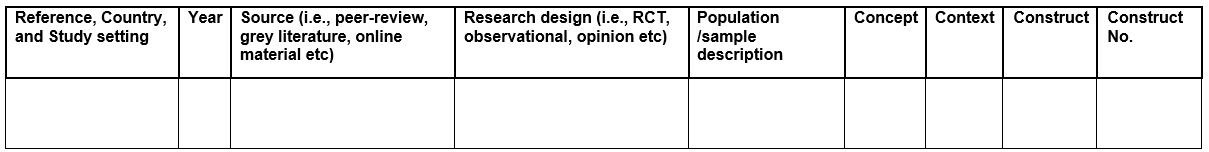
**

Supplement: Supplementary file 1 — Additional file 1. Standardized Charting Form. [file 12910_2022_849_MOESM1_ESM.docx]
