# Supplementary material for: Electronic informed consent criteria for research ethics review: a scoping review
Source: BMC Med Ethics. 2022 Nov 21;23:117. doi: 10.1186/s12910-022-00849-x (PMC9682656; doi:10.1186/s12910-022-00849-x)
Supplement: Supplementary file 2 — Additional file 2. Search strategy for the databases. [file 12910_2022_849_MOESM2_ESM.docx]

# Appendix II

Search strategy for the databases

**PubMed:**

| **No.** | **Concept** | **Search string** | **Number** |
| --- | --- | --- | --- |
| #1 | Consent | "Informed Consent"[Mesh] OR  "Consent Forms"[Mesh] OR  "Comprehension"[Mesh] OR  Consent[ti] | 57,445 |
| #2 | Digital | "Electronic Health Records"[Mesh] OR  "electronics"[Mesh] OR  "Technology"[Mesh]  "telemedicine”[Mesh]  digital[ti] OR  Electronic[ti] OR  Online[ti] OR  Web[ti] OR  Audio-visual[ti] | 104,426 |
| #3 |  | (1 AND 2) | 656 |
| #4 |  | (1 AND 2) OR (econsent OR e-consent OR teleconsent) | 719 |

**SCOPUS:**

| **No.** | **Concept** | **Search string** | **Number** |
| --- | --- | --- | --- |
| #1 | Consent | "Informed Consent"[Mesh] OR  "Consent Forms"[Mesh] OR  "Comprehension"[Mesh] | [243](https://pubmed.ncbi.nlm.nih.gov/?term=%22Informed+Consent%22%5BMesh%5D+OR+%0A%22Consent+Forms%22%5BMesh%5D+OR+%0A%22Comprehension%22%5BMesh%5D+OR%0AConsent%5Bti%5D&sort=relevance&ac=no) |
| #2 | Digital | "Electronic Health Records"[Mesh] OR  "electronics"[Mesh] OR  "Technology"[Mesh]  "telemedicine”[Mesh] | 93 |
| #3 |  | (1 AND 2) | 74 |
| #4 |  | (1 AND 2) OR (econsent OR e-consent OR teleconsent) | 71 |

**EBSCO:**

| **No.** | **Concept** | **Search string** | **Number** |
| --- | --- | --- | --- |
| #1 | Consent | "Informed Consent"[Mesh] OR  "Consent Forms"[Mesh] OR  "Comprehension"[Mesh] | [15,933](https://pubmed.ncbi.nlm.nih.gov/?term=%22Informed+Consent%22%5BMesh%5D+OR+%0A%22Consent+Forms%22%5BMesh%5D+OR+%0A%22Comprehension%22%5BMesh%5D+OR%0AConsent%5Bti%5D&sort=relevance&ac=no) |
| #2 | Digital | "Electronic Health Records"[Mesh] OR  "electronics"[Mesh] OR  "Technology"[Mesh]  "telemedicine”[Mesh] | 5,971 |
| #3 |  | (1 AND 2) | 794 |
| #4 |  | (1 AND 2) OR (econsent OR e-consent OR teleconsent) | 1087 |
