# Supplementary material for: Electronic informed consent criteria for research ethics review: a scoping review
Source: BMC Med Ethics. 2022 Nov 21;23:117. doi: 10.1186/s12910-022-00849-x (PMC9682656; doi:10.1186/s12910-022-00849-x)
Supplement: Supplementary file 3 — Additional file 3. Data Collection Table. [file 12910_2022_849_MOESM3_ESM.docx]

# Appendix III

Data Collection Table

| **No.** | **Author/Year** | **Title** | **Geographic Area** | **Article Type** | **Study Design** | **Population** | **Page No.** | **Item No.** | **Item** | **Summarized description** | **Concept** | **Code** |
| --- | --- | --- | --- | --- | --- | --- | --- | --- | --- | --- | --- | --- |
| 1 | (Bergmann et al. 2005) | An eConsent-based System Architecture Supporting Cooperation in Integrated Healthcare Networks | Europe | Perspective | Methodical/Framework Analysis | Electronic Health Record | 965 | 1.1 | The patient is always able to control and restrict the access to the shared record | Participant is always able to control and restrict the access to the shared record | Autonomy | f |
| 2 | (Brooks et al. 2016) | An electronic surgical order, undertaking patient education, and obtaining informed consent for regional analgesia before the day of surgery reduce block-related delays | Europe | Original Research | Randomised Controlled Trial | Regional Anesthesia | 63 | 2.1 | increasing the patient-centered nature of informed consent for procedures | Participant is always able to control and restrict the access to the shared record | Autonomy | f |
|  |  |  |  |  |  |  |  | 2.2 | Undertaking the consent process before the day intervention may be better, as it gives patients more time to reflect and to ask questions, as well as to consider their options | Undertaking the consent process before the day intervention | Comprehension Engagement | c |
|  |  |  |  |  |  |  |  | 2.3 | identifying and reducing learning and language barriers in the informed consent process | Simplify the language | Language | l |
|  |  |  |  |  |  |  |  | 2.4 | optimizing preoperative patient education and health literacy | Undertaking the consent process before the day intervention | Comprehension Engagement | c |
|  |  |  |  |  |  |  |  | 2.5 | implementing culturally sensitive and language-specific, tablet-based decision support tools | Simplify the language | Language | l |
| 3 | (Chantler et al. 2020) | Does electronic consent improve the logistics and uptake of HPV vaccination in adolescent girls? A mixed- methods theory informed evaluation of a pilot intervention | Europe | Original Research | Randomised Controlled Trial | Uptake of HPV Vaccination | 8 | 3.1 | e- consent intervention inadvertently bypassed some adolescents’ information needs and related opportunity to talk to their parents about HPV vaccination | Give opportunity to gather information from parents/LAR | Parental Consent | p |
| 4 | (Chen et al. 2020) | Replacing Paper Informed Consent with Electronic Informed Consent for Research in Academic Medical Centers: A Scoping Review | North America | Review | Scoping Review | Research in Academic Medical Centers | 82 | 4.1 | Younger users were found to be more satisfied with using technology for informed consent. Conversely, older participants may be more skeptical of mobile technology and thus more concerned about their privacy and confidentiality while using their phones | Identify the demographics and needs of the participants | Accessibility | a |
|  |  |  |  |  |  |  | 83 | 4.2 | minorities and rural area residents expressed concern over access to computers and the internet, computer literacy, privacy and confidentiality, relating to the use of eConsent for research | Identify the demographics and needs of the participants | Accessibility | a |
|  |  |  |  |  |  |  |  | 4.3 | the need to link the samples with participants’ electronic medical record (EMR), and other information such as family history, lifestyle and environmental risk factors; and the requirement that participants must broadly consent to any type of research | Identify the demographics and needs of the participants | Accessibility | a |
|  |  |  |  |  |  |  |  | 4.4 | biobanks typically operate under different protocols approved by various IRBs; separate consents may be needed and a way of tracking them is essential | Establish tracking mechanism for separate consents | Accessibility | a |
|  |  |  |  |  |  |  |  | 4.5 | using technology to allow participants to monitor and keep track of use of their biological samples over time | Establish digital technology for participants to track the use of their biological sample over time | Digital Content | d |
|  |  |  |  |  |  |  |  | 4.6 | electronic signature must be unique to one individual, and organizations must verify the identity of the individual | Use validated electronic signature | Digital Content | d |
|  |  |  |  |  |  |  |  | 4.7 | To secure the participants’ information, researchers have used encryption for data in transit, not storing information on a server | Use encryption for data in transit | Confidentiality | y |
|  |  |  |  |  |  |  |  | 4.8 | concerns about storing biometric information on cloud storage and suggested storing the information on the users’ local devices instead | Establish digital storing biometric information on cloud storage | Digital Content | d |
|  |  |  |  |  |  |  |  | 4.9 | pseudonymize the data | Use encryption for data in transit | Confidentiality | y |
|  |  |  |  |  |  |  |  | 4.10 | research participants have the right to determine how and with whom their medical information is shared | Participant is always able to control and restrict the access to the shared record | Autonomy | f |
|  |  |  |  |  |  |  |  | 4.11 | Implicit consent or research requiring opting-out is not acceptable informed consent | Use only explicit consent | Autonomy | f |
|  |  |  |  |  |  |  |  | 4.12 | the benefit of social annotation, where participants can see each other’s comments about the eConsent, as a trusted way to question the research while lessening social pressure | Support social annotation whereby participants can see each other comments for discussions | Comprehension Engagement | c |
|  |  |  |  |  |  |  |  | 4.13 | participants to complete the consent form in real-time with remote researchers to be interactive and similar to the in-person experience | Provide interactive personnel to optimize comprehension and trust | Comprehension Engagement | c |
|  |  |  |  |  |  |  | 84 | 4.14 | e-consent had interactive components, quizzes, tailored information, graphical media, and annotations | Support interactive digital components such as online quiz, graphical media and audio-visual aid | Digital Content | d |
|  |  |  |  |  |  |  |  | 4.15 | in-person interactions or other forms of communications with researchers remain a part of the eConsent process, to ensure participants’ understanding of consent information and to foster trust, particularly for more complex and riskier studies | Provide interactive personnel to optimize comprehension and trust | Comprehension Engagement | c |
|  |  |  |  |  |  |  |  | 4.16 | when participants were given the choice to receive more, the same, or less information in the eConsent form than presented on a paper consent form, a majority of them choose less information, suggesting that the amount of information in the paper consent was more detailed than what participants are willing to read | Provide interactive personnel to optimize comprehension and trust | Comprehension Engagement | c |
|  |  |  |  |  |  |  |  | 4.17 | Many participants think less than half of the consent form is vital, and perceive the consent form as providing legal protection for researchers, rather than informing participants | Use only explicit consent | Autonomy | f |
|  |  |  |  |  |  |  |  | 4.18 | use of eConsent to be less stressful compared to paper because they could proceed at their own pace and have more control over the consent process | Participant is always able to control their own pace during consent process | Autonomy | f |
|  |  |  |  |  |  |  |  | 4.19 | emphasized benefits of integrating research consent data with electronic health record (EHR) systems and electronic data warehouses | Integrate research consent data with electronic health record | Accessibility | a |
|  |  |  |  |  |  |  |  | 4.20 | to consider ontological models of consent | Integrate research consent data with electronic health record | Accessibility | a |
|  |  |  |  |  |  |  |  | 4.21 | the need for information technology infrastructure, including wifi connectivity, informatics personnel for development, and helpdesk personnel for support | Support information technology infrastructure | Digital Content | d |
|  |  |  |  |  |  |  |  | 4.22 | need for proper authentication through usernames and passwords, finger-drawn signatures, biometrics, and blockchain | Provide secured and proper authentication | Confidentiality | y |
|  |  |  |  |  |  |  |  | 4.23 | USign, a signature verification method that can integrate with existing eConsent systems and provide a new authentication token | Use validated electronic signature | Digital Content | d |
|  |  |  |  |  |  |  |  | 4.24 | approaches to tracking versions of protocols and informed consent forms to which participants consented | Establish tracking mechanism for separate consents | Accessibility | a |
|  |  |  |  |  |  |  | 85 | 4.25 | Changes to consent range from protocol updates and amendments to withdrawal | Establish tracking mechanism for separate consents | Accessibility | a |
|  |  |  |  |  |  |  |  | 4.26 | dynamic consent model that allows for personalization and flexibility, such as allowing a biobank participant to amend their broad consent based on new research activities | Allow participant to amend their consent | Autonomy | f |
|  |  |  |  |  |  |  |  | 4.27 | identifying the organization type of the data recipient as participants may only wish to consent to providing limited data to for-profit organizations | Allow participant to amend their consent | Autonomy | f |
| 5 | (Chen et al. 2019) | Evaluation of a REDCap-based Workflow for Supporting Federal Guidance for Electronic Informed Consent | North America | Perspective | Methodical/Framework Analysis | REDCap-based Workflow | 169 | 5.1 | Researchers are able to successfully enroll participants | Integrate research consent data with electronic health record | Accessibility | a |
|  |  |  |  |  |  |  |  | 5.2 | Participants receive a copy of the completed consent form | Participants receive a copy of the completed consent form | Accessibility | a |
|  |  |  |  |  |  |  |  | 5.3 | Participants are able to update elections | Allow participant to amend their consent | Autonomy | f |
|  |  |  |  |  |  |  |  | 5.4 | Participants are able to withdraw | Allow participant to amend their consent | Autonomy | f |
|  |  |  |  |  |  |  |  | 5.5 | IRB amendments are successfully implemented | Allow IRB oversight and amendment | Accessibility | a |
|  |  |  |  |  |  |  |  | 5.6 | eConsent complies with local guidance (e.g., security) | Provide secured and proper authentication | Confidentiality | y |
|  |  |  |  |  |  |  |  | 5.7 | eConsent complies with federal guidance (e.g.,HIPAA) | Provide secured and proper authentication | Confidentiality | y |
|  |  |  |  |  |  |  |  | 5.8 | eConsent must contain everything required by 45 CFR 46.116 (General requirements for informed consent) and 21 CFR 50.25 (Elements of informed consent) |  |  |  |
|  |  |  |  |  |  |  |  | 5.9 | eConsent must be easy to navigate, allowing user to go forward and back and to stop and continue at a later time | Participant is always able to control their own pace during consent process | Autonomy | f |
|  |  |  |  |  |  |  |  | 5.10 | Subjects should have the option to use paper-based or electronic IC methods completely or partially throughout the process | Allow participant to amend their consent | Autonomy | f |
|  |  |  |  |  |  |  |  | 5.11 | Process must be conducted at study site or remotely. If remote, must include mechanism to ensure person electronically signing is the person participating or their LAR | Use validated electronic signature | Digital Content | d |
|  |  |  |  |  |  |  |  | 5.12 | eConsent must be legally binding, regardless of onsite or off-site | Needs to be legally binding | Confidentiality | y |
|  |  |  |  |  |  |  |  | 5.13 | eConsent must include mechanism to ensure person electronically signing is the participant or their LAR | Use validated electronic signature | Digital Content | d |
|  |  |  |  |  |  |  |  | 5.14 | Questions should be answered via in person discussions or combination of electronic messaging, telephone calls, video conferencing, or live chat with remotely located investigator or study personnel | Provide interactive personnel to optimize comprehension and trust | Comprehension Engagement | c |
|  |  |  |  |  |  |  |  | 5.15 | eConsent should have methods in place to ensure the process allows subjects the opportunity to consider whether or not to participate and to ask questions | Allow participant to amend their consent | Autonomy | f |
|  |  |  |  |  |  |  |  | 5.16 | When live chat or video is used, there should be reminders to conduct the discussion in a private location | Provide options to discuss in private | Confidentiality | y |
|  |  |  |  |  |  |  |  | 5.17 | Subjects should be given a description of how and when they will receive answers to their questions, and how they can communicate back in the event that they sustain a research-related injury | Support social annotation whereby participants can see each other comments for discussions | Comprehension Engagement | c |
|  |  |  |  |  |  |  |  | 5.18 | eConsent content should be appropriate for intended audience based on age, language, and comprehension level | Identify the demographics and needs of the participants | Accessibility | a |
|  |  |  |  |  |  |  |  | 5.19 | eConsent must contain a statement that significant new findings or updates/amendements that may affect the subject's willingness to continue will be communicated to the subject or their LAR and that they'll have the opportunity to ask questions and sign an updated eConsent | Allow participant to amend their consent | Autonomy | f |
|  |  |  |  |  |  |  |  | 5.20 | eConsent must comply with all 21 CFR 11 (Electronic Records; Electronic Signatures) requirements |  |  |  |
|  |  |  |  |  |  |  |  | 5.21 | eConsent must comply with 21 CFR 11 (Electronic Records; Electronic Signatures) |  |  |  |
|  |  |  |  |  |  |  |  | 5.22 | eConsent must comply with 45 CFR 46 (General requirements for informed consent) and 21 CFR 50 (Elements of informed consent) |  |  |  |
|  |  |  |  |  |  |  |  | 5.23 | IRB must determine that there are sufficient provisions for soliciting the assent of children | Give opportunity to gather information from parents/LAR | Parental Consent | p |
|  |  |  |  |  |  |  |  | 5.24 | Subjects must be given a copy of of the signed informed consent form unless requirement for documentation has been waived | Participants receive a copy of the completed consent form | Accessibility | a |
|  |  |  |  |  |  |  |  | 5.25 | If the eConsent uses multimedia to convey information related to research, hyperlinks should be provided on printed paper copies and accessible until study completion | Support interactive digital components such as online quiz, graphical media and audio-visual aid | Digital Content | d |
|  |  |  |  |  |  |  |  | 5.26 | eConsent must be secure with restricted access as per 21 CFR 11 and should include methods to ensure confidentiality regarding identity, subject participation, and personal information after IC has been obtained | Provide secured and proper authentication | Confidentiality | y |
|  |  |  |  |  |  |  | 170 | 5.27 | If the entity holding PII is a covered entity or BAA of one, eConsent must adhere to HIPAA rules regarding privacy, security, and breach notifications | Provide secured and proper authentication | Confidentiality | y |
|  |  |  |  |  |  |  |  | 5.28 | When covered entities seek authorization forms, they must provide the participant with a copy of the signed authorization form | Participants receive a copy of the completed consent form | Accessibility | a |
|  |  |  |  |  |  |  |  | 5.29 | All forms, electronic and paper, must be submitted to the IRB, along with any modifications | Allow IRB oversight and amendment | Accessibility | a |
|  |  |  |  |  |  |  |  | 5.30 | Investigators should discuss plans for using eConsent with IRB before finalizing development | Allow IRB oversight and amendment | Accessibility | a |
|  |  |  |  |  |  |  |  | 5.31 | IRB must review and have authority to approve, require modifications to, or disapprove all research activities. IRB must approve and review eConsent and any amendments | Allow IRB oversight and amendment | Accessibility | a |
|  |  |  |  |  |  |  |  | 5.32 | IRB must approve and review eConsent and any amendments, as well as maintain/retain copies of any materials | Allow IRB oversight and amendment | Accessibility | a |
|  |  |  |  |  |  |  |  | 5.33 | IRB should review any optional questions or methods used to gauge subject comprehension and ensure that eConsent materials are usable, as well as maintain copies of any study-related information | Allow IRB oversight and amendment | Accessibility | a |
|  |  |  |  |  |  |  |  | 5.34 | eConsent documentation must be available for federal review for INDs. IDEs must include eConsent documentation. Documentation must be the same materials that will be presented to subjects | Allow IRB oversight and amendment | Accessibility | a |
|  |  |  |  |  |  |  |  | 5.35 | eConsent should incorporate procedures to ensure documents can be archived appropriate and retrieved easily in compliance with applicable FDA regulations | Integrate research consent data with electronic health record | Accessibility | a |
|  |  |  |  |  |  |  |  | 5.36 | When sites are inspected by FDA, FDA must be granted access to sitespecific versions of the EIC along with all amendments and subject-signed forms | Allow IRB oversight and amendment | Accessibility | a |
| 6 | (De Sutter et al. 2020) | Implementation of Electronic Informed Consent in Biomedical Research and Stakeholders’ Perspectives: Systematic Review | Europe | Review | Systematic Review | Biomedical Research and Stakeholders’ Perspectives | 9 | 6.1 | Implement audio, video, graphics (ie, icons, progress bar) and hyperlinks | Support interactive digital components such as online quiz, graphical media and audio-visual aid | Digital Content | d |
|  |  |  |  |  |  |  |  | 6.2 | Use simple, concise language and implement a bullet point format | Simplify the language | Language | l |
|  |  |  |  |  |  |  |  | 6.3 | Give the possibility to research participants to highlight information that is difficult to understand in order to facilitate the discussion with the research staff | Support social annotation whereby participants can see each other comments for discussions | Comprehension Engagement | c |
|  |  |  |  |  |  |  |  | 6.4 | Depending on the research study, make electronic informed consent available in multiple languages by using subtitles or translated text | Use multiple language | Language | l |
|  |  |  |  |  |  |  |  | 6.5 | Implement a quality assurance process to check the input of the end user | Establish digital storing biometric information on cloud storage | Digital Content | d |
|  |  |  |  |  |  |  |  | 6.6 | Pay attention to the personal connection between the research participants and the research staff | Provide interactive personnel to optimize comprehension and trust | Comprehension Engagement | c |
|  |  |  |  |  |  |  |  | 6.7 | Implement quizzes to assess the participants’ level of comprehension | Support interactive digital components such as online quiz, graphical media and audio-visual aid | Digital Content | d |
|  |  |  |  |  |  |  |  | 6.8 | Give the possibility to review information by using interactive technology or the printed electronic informed consent form | Support interactive digital components such as online quiz, graphical media and audio-visual aid | Digital Content | d |
|  |  |  |  |  |  |  |  | 6.9 | Guarantee adequate support for people with limited computer literacy, visual/auditory impairment and people who do not have access to internet or computers | Support information technology infrastructure | Accessibility | a |
|  |  |  |  |  |  |  |  | 6.10 | Collaborate with health authorities and ethics committees to create a framework for reviewing and implementing electronic informed consent | Allow IRB oversight and amendment | Accessibility | a |
|  |  |  |  |  |  |  |  | 6.11 | Implement controlled access systems for several stakeholder groups and pay attention to a secured electronic informed consent platform. Note: provide sufficient information to potential research participants about privacy aspects of the platform | Provide secured and proper authentication | Confidentiality | y |
|  |  |  |  |  |  |  |  | 6.12 | Make sure that the secure transfer of files is possible between stakeholder groups | Use encryption for data in transit | Confidentiality | y |
|  |  |  |  |  |  |  |  | 6.13 | Provide online storage of the informed consent | Establish digital storing biometric information on cloud storage | Digital Content | d |
|  |  |  |  |  |  |  |  | 6.14 | Support online withdrawal with documentation of the reasons for withdrawal | Allow participant to amend their consent | Autonomy | f |
|  |  |  |  |  |  |  |  | 6.15 | Pay attention to transparency regarding the use of participants’health information and their right to control the sharing and use of this information | Participant is always able to control and restrict the access to the shared record | Autonomy | f |
|  |  |  |  |  |  |  |  | 6.16 | Implement the possibility to update research participants frequently with information about preliminary results, followup studies and main outcomes | Allow participant to amend their consent | Autonomy | f |
|  |  |  |  |  |  |  |  | 6.17 | Implement definitions | Support information technology infrastructure | Digital Content | d |
|  |  |  |  |  |  |  |  | 6.18 | Implement social annotations but mind the emotional force | Support social annotation whereby participants can see each other comments for discussions | Comprehension Engagement | c |
|  |  |  |  |  |  |  |  | 6.19 | Implement a personalized approach | Provide interactive personnel to optimize comprehension and trust | Comprehension Engagement | c |
| 7 | (Faden et al. 2013) | Ethics and Informed Consent for Comparative Effectiveness Research With Prospective Electronic Clinical Data | North America | Perspective | Methodical/Framework Analysis | Electronic Health Record | 53 | 7.1 | patients are regularly informed of the health care institution’s commitment to learning through the integration of research and practice | Provide interactive personnel to optimize comprehension and trust | Comprehension Engagement | c |
|  |  |  |  |  |  |  |  | 7.2 | there are appropriate protections for patients’ rights and interests | Provide secured and proper authentication | Confidentiality | y |
| 8 | (Frost et al. 2021) | Electronic informed consent information for residual newborn specimen research: findings from focus groups with diverse populations | North America | Perspective | Interviews/Surveys | App Development | 202 | 8.1 | prefer to use the video rather than the app because the visuals in the video contributed to their understanding more than the text in the app | Support interactive digital components such as online quiz, graphical media and audio-visual aid | Digital Content | d |
|  |  |  |  |  |  |  |  | 8.2 | a video tool in the appropriate language would be very useful for educational purposes | Simplify the language | Language | l |
|  |  |  |  |  |  |  | 201 | 8.3 | more people from diverse backgrounds should be seen in the videos | Identify the demographics and needs of the participants | Accessibility | a |
| 9 | (Hall et al. 2017) | Use of Videos Improves Informed Consent Comprehension in Web-Based Surveys Among Internet-Using Men Who Have Sex With Men: A Randomized Controlled Trial | North America | Original Research | Randomised Controlled Trial | Web-based HIV Research with MSM | 5 | 9.1 | a deduplication protocol was determined before data collection to remove duplicate or artificial survey attempts | Use encryption for data in transit | Confidentiality | y |
|  |  |  |  |  |  |  |  | 9.2 | indicators of potential fraudulent responses from artificial hacking or bot programs possibly aimed at getting the monetary incentive | Provide secured and proper authentication | Confidentiality | y |
|  |  |  |  |  |  |  |  | 9.3 | put measures in place including Completely Automated Public Turing test to tell Computers and Humans Apart (CAPTCHA) codes and verification of email addresses submitted for incentives | Provide secured and proper authentication | Confidentiality | y |
|  |  |  |  |  |  |  | 4 | 9.4 | time spent on consent was measured by amount of time the respondent stayed on the Web page with the consent information | Participant is always able to control their own pace during consent process | Autonomy | f |
|  |  |  |  |  |  |  |  | 9.5 | in order to progress to the next page, respondents had to click on a button indicating they agree (or do not agree) to participate in the study | Establish tracking mechanism for separate consents | Accessibility | a |
| 10 | (Haussen et al. 2020) | Legal authorized representative experience with smartphone- based electronic informed consent in an acute stroke trial | North America | Original Research | Randomised Controlled Trial | Acute Stroke Trial | 483 | 10.1 | survey project located on a static URL that can be remotely accessed (via smartphone browser) | Establish digital storing biometric information on cloud storage | Digital Content | d |
|  |  |  |  |  |  |  |  | 10.2 | establishing time interval between patient arrival at our institution and consent being electronically signed by the LAR | Use validated electronic signature | Digital Content | d |
| 11 | (Haussen et al. 2017) | Utilization of a Smartphone Platform for Electronic Informed Consent in Acute Stroke Trials | North America | Original Research | Randomised Controlled Trial | Acute Stroke Trial | 3157 | 11.1 | a standard link to the URL is sent via text message and/or email to the LAR, generating a primary instrument (e-Consent form) | Establish digital storing biometric information on cloud storage | Digital Content | d |
|  |  |  |  |  |  |  |  | 11.2 | entering the LAR’s email address (for a copy of the consent) and names of the patient and LAR, selecting relationship status, and signing freehand | Integrate research consent data with electronic health record | Accessibility | a |
|  |  |  |  |  |  |  |  | 11.3 | a record/record ID are automatically created in the database and an e-Consent Process Attestation form autogenerated, which is later completed by the physician who obtained the consent | Integrate research consent data with electronic health record | Accessibility | a |
| 12 | (Hwang et al. 2015) | Description of a Mobile-based Electronic Informed Consent System Development | Asia | Perspective | Methodical/Framework Analysis | App Development | 897 | 12.1 | included drawing features | Support interactive digital components such as online quiz, graphical media and audio-visual aid | Digital Content | d |
|  |  |  |  |  |  |  |  | 12.2 | supported certified electronic signature modules | Use validated electronic signature | Digital Content | d |
|  |  |  |  |  |  |  |  | 12.3 | prevented forgery by saving captured images | Use encryption for data in transit | Confidentiality | y |
| 13 | (Hyeoneui et al. 2017) | iCONCUR: informed consent for clinical data and bio-sample use for research | North America | Original Research | Interviews/Surveys | App Development | 381 | 13.1 | included information on how to use the iCONCUR tool: creating an account, indicating data sharing preferences, and reviewing data usage | Integrate research consent data with electronic health record | Accessibility | a |
|  |  |  |  |  |  |  |  | 13.2 | modify their (participants) preferences as frequently as they wanted | Allow participant to amend their consent | Autonomy | f |
|  |  |  |  |  |  |  |  | 13.3 | research staff member was available via email and phone to answer any questions on using iCONCUR during the study period | Provide interactive personnel to optimize comprehension and trust | Comprehension Engagement | c |
|  |  |  |  |  |  |  | 382 | 13.4 | database analyst double-checked all patient preferences to make sure each choice was honored | Establish tracking mechanism for separate consents | Accessibility | a |
| 14 | (Issa et al. 2006) | Informed Versus Uninformed Consent for Prostate Surgery: The Value of Electronic Consents | North America | Original Research | Randomised Controlled Trial | Prostate Surgery | 695 | 14.1 | displayed on a computer | Support interactive digital components such as online quiz, graphical media and audio-visual aid | Digital Content | d |
|  |  |  |  |  |  |  |  | 14.2 | populated with a description of the procedure as well as with the pertinent risks, benefits and alternatives | Fulfill general requirements and elements of informed consent | Comprehension Engagement | c |
|  |  |  |  |  |  |  |  | 14.3 | Additional information and risks specific to the individual can be added or deleted by the provider as needed | Establish digital storing biometric information on cloud storage | Digital Content | d |
|  |  |  |  |  |  |  |  | 14.4 | The patient, provider and a witness sign an electronic signature pad | Use validated electronic signature | Digital Content | d |
|  |  |  |  |  |  |  |  | 14.5 | The completed consent is stored in the patient electronic medical record and a paper copy is given to the patient | Participants receive a copy of the completed consent form | Accessibility | a |
| 15 | (Jimison et al. 1998) | The Use of Multimedia in the Informed Consent Process | North America | Original Research | Interviews/Surveys | App Development | 249 | 15.1 | Make the consent forms simpler and shorter | Simplify the language | Language | l |
|  |  |  |  |  |  |  |  | 15.2 | Provide a summary of highlights, with the details kept separate | Establish tracking mechanism for separate consents | Accessibility | a |
|  |  |  |  |  |  |  |  | 15.3 | Provide a glossary; define terms | Support information technology infrastructure | Digital Content | d |
|  |  |  |  |  |  |  |  | 15.4 | Use lay language throughout | Simplify the language | Language | l |
|  |  |  |  |  |  |  |  | 15.5 | Provide information on clinical trials in general | Fulfill general requirements and elements of informed consent | Comprehension Engagement | c |
|  |  |  |  |  |  |  |  | 15.6 | Provide space to write questions | Support social annotation whereby participants can see each other comments for discussions | Comprehension Engagement | c |
|  |  |  |  |  |  |  |  | 15.7 | Use larger font for text | Support interactive digital components such as online quiz, graphical media and audio-visual aid | Digital Content | d |
|  |  |  |  |  |  |  |  | 15.8 | Emphasize what is important | Provide interactive personnel to optimize comprehension and trust | Comprehension Engagement | c |
|  |  |  |  |  |  |  |  | 15.9 | Use graphics and video | Support interactive digital components such as online quiz, graphical media and audio-visual aid | Digital Content | d |
| 16 | (Kluge 2004) | Informed consent and the security of the electronic health record (EHR): some policy considerations | North America | Perspective | Methodical/Framework Analysis | Electronic Health Record | 229 | 16.1 | potential subjects of electronic health records (EHRs) should be made aware of the existence of any systems, programs or devices for collecting and/or communicating data about them | Integrate research consent data with electronic health record | Accessibility | a |
|  |  |  |  |  |  |  |  | 16.2 | potential subjects have a right to the actual construction of such records as well as to the use, storage, communication, manipulation and other processing of these records and of the data that are contained in them | Participant is always able to control and restrict the access to the shared record | Autonomy | f |
| 17 | (C. Lawrence et al. 2020a) | A REDCap-based model for electronic consent (eConsent): Moving toward a more personalized consent | North America | Original Research | Methodical/Framework Analysis | App Development | 349 | 17.1 | Use animation for invasive procedures | Support interactive digital components such as online quiz, graphical media and audio-visual aid | Digital Content | d |
|  |  |  |  |  |  |  |  | 17.2 | Videos should be short, informative, but clear and concise | Support interactive digital components such as online quiz, graphical media and audio-visual aid | Digital Content | d |
|  |  |  |  |  |  |  |  | 17.3 | Show diversity of ages and race/ethnicity | Identify the demographics and needs of the participants | Accessibility | a |
|  |  |  |  |  |  |  |  | 17.4 | Use videos to tell a story (e.g., greeting by nurse, inserting IV) | Support interactive digital components such as online quiz, graphical media and audio-visual aid | Digital Content | d |
|  |  |  |  |  |  |  |  | 17.5 | Include subtitles for Spanish-speaking audience | Use multiple language | Language | l |
|  |  |  |  |  |  |  |  | 17.6 | Use high-quality filmmaking | Support interactive digital components such as online quiz, graphical media and audio-visual aid | Digital Content | d |
|  |  |  |  |  |  |  |  | 17.7 | Avoid voices which sound computer generated | Support interactive digital components such as online quiz, graphical media and audio-visual aid | Digital Content | d |
|  |  |  |  |  |  |  |  | 17.8 | Avoid 3D animation and cartoon-like 2D animation | Support interactive digital components such as online quiz, graphical media and audio-visual aid | Digital Content | d |
|  |  |  |  |  |  |  |  | 17.9 | Avatars should appear as medical staff (e.g., lab coats) | Support interactive digital components such as online quiz, graphical media and audio-visual aid | Digital Content | d |
|  |  |  |  |  |  |  |  | 17.10 | Avatar should not replace interaction with study staff | Provide interactive personnel to optimize comprehension and trust | Comprehension Engagement | c |
|  |  |  |  |  |  |  |  | 17.11 | Avatar helpful if consent sent out in advance | Undertaking the consent process before the day intervention | Comprehension Engagement | c |
|  |  |  |  |  |  |  |  | 17.12 | Train study staff to be patient, supportive, and knowledgeable | Provide interactive personnel to optimize comprehension and trust | Comprehension Engagement | c |
|  |  |  |  |  |  |  |  | 17.13 | Use clear and concise language in consent documents | Simplify the language | Language | l |
|  |  |  |  |  |  |  |  | 17.14 | Partner with local organizations | Allow IRB oversight and amendment | Accessibility | a |
|  |  |  |  |  |  |  |  | 17.15 | Increase diversity of study staff | Provide interactive personnel to optimize comprehension and trust | Comprehension Engagement | c |
|  |  |  |  |  |  |  |  | 17.16 | Focus on value of research to the individual and future generations | Fulfill general requirements and elements of informed consent | Comprehension Engagement | c |
|  |  |  |  |  |  |  |  | 17.17 | Address fears and lack of trust openly by incorporating testimonials | Support social annotation whereby participants can see each other comments for discussions | Comprehension Engagement | c |
|  |  |  |  |  |  |  |  | 17.18 | Provide option of paper consent to participants | Allow participant to amend their consent | Autonomy | f |
|  |  |  |  |  |  |  |  | 17.19 | Consider breaking up sections by “pages” | Support interactive digital components such as online quiz, graphical media and audio-visual aid | Digital Content | d |
|  |  |  |  |  |  |  |  | 17.20 | Videos were representative, appropriate, and informative | Support interactive digital components such as online quiz, graphical media and audio-visual aid | Digital Content | d |
|  |  |  |  |  |  |  |  | 17.21 | Provide both paper and eConsent options | Allow participant to amend their consent | Autonomy | f |
|  |  |  |  |  |  |  |  | 17.22 | Useful if potentially can review in advance of consenting | Undertaking the consent process before the day intervention | Comprehension Engagement | c |
|  |  |  |  |  |  |  |  | 17.23 | Include additional training components including use of social media, building empathy, and addressing data privacy issues | Provide options to discuss in private | Confidentiality | y |
|  |  |  |  |  |  |  |  | 17.24 | Offer tiers of training based on recruiter experience | Provide interactive personnel to optimize comprehension and trust | Comprehension Engagement | c |
|  |  |  |  |  |  |  |  | 17.25 | Develop interactive online training | Support interactive digital components such as online quiz, graphical media and audio-visual aid | Digital Content | d |
|  |  |  |  |  |  |  |  | 17.26 | eConsent should not replace interaction with study staff | Provide interactive personnel to optimize comprehension and trust | Comprehension Engagement | c |
|  |  |  |  |  |  |  |  | 17.27 | Offer choice regarding eConsent add-ons (e.g., videos should be optional) | Participant is always able to control and restrict the access to the shared record | Autonomy | f |
|  |  |  |  |  |  |  |  | 17.28 | Consider utilizing features to accommodate dialects (e.g., hover over to show differing translations for a word) | Use multiple language | Language | l |
| 18 | (Lee 2017) | Ethics and subsequent use of electronic health record data | North America | Perspective | Methodical/Framework Analysis | Electronic Health Record | 145 | 18.1 | ensure that data and results are valid, and demonstrate usefulness | Undertaking the consent process before the day of intervention | Comprehension Engagement | c |
|  |  |  |  |  |  |  |  | 18.2 | implement data protections to reduce the risk of unauthorized disclosure of personal health and healthbehavior information | Use encryption for data in transit | Confidentiality | y |
| 19 | (Lindegren et al. 2021) | An evaluation of three designs to engage users when providing their consent on smartphones | Europe | Original Research | Interviews/Surveys | Electronic Health Record | 404 | 19.1 | did not use sensitive or actual data without anonymisation | Use encryption for data in transit | Confidentiality | y |
|  |  |  |  |  |  |  | 405 | 19.2 | Icons can be used to display logical arrangements of the interface to ensure awareness in privacy systems | Provide secured and proper authentication | Confidentiality | y |
|  |  |  |  |  |  |  | 406 | 19.3 | Tutorials are important in order to make users aware, feel in control and comprehend how information is handled | Support social annotation whereby participants can see each other comments for discussions | Comprehension Engagement | c |
|  |  |  |  |  |  |  | 406 | 19.4 | Drag and Drop (DAD) concept to be used in consent forms to avoid users’ automated behaviours that stemmed from dialogue boxes with two alternatives of ‘OK’ and ‘Cancel’ | Support interactive digital components such as online quiz, graphical media and audio-visual aid | Digital Content | d |
| 20 | (Phillippi et al. 2018) | Electronic Informed Consent to Facilitate Recruitment of Pregnant Women into Research | North America | Original Research | Randomised Controlled Trial | Recruitment of Pregnant Women into Research | 3 | 20.1 | secure portal, preferably web-based, for maximum availability | Provide secured and proper authentication | Confidentiality | y |
|  |  |  |  |  |  |  |  | 20.2 | allows secure digital completion of consent forms, including participant signature, using a touch screen or mouse | Provide secured and proper authentication | Confidentiality | y |
|  |  |  |  |  |  |  |  | 20.3 | Signed forms can be downloaded or printed | Use validated electronic signature | Digital Content | d |
|  |  |  |  |  |  |  |  | 20.4 | study documents can be provided to and obtained from participants then shared among research staff, auditors, monitors, and sponsors using similar portal | Integrate research consent data with electronic health record | Accessibility | a |
|  |  |  |  |  |  |  | 4 | 20.5 | all response transmissions were encrypted for security | Use encryption for data in transit | Confidentiality | y |
|  |  |  |  |  |  |  |  | 20.6 | the investigator offered to remain on the phone to answer questions while the woman completed the consent process | Provide interactive personnel to optimize comprehension and trust | Comprehension Engagement | c |
|  |  |  |  |  |  |  |  | 20.7 | could complete the consent later if she did not want or was not able to complete the process at that time | Participant is always able to control their own pace during consent process | Autonomy | f |
|  |  |  |  |  |  |  |  | 20.8 | could add her name and age in text boxes and signed the consent form using her mouse or finger | Use validated electronic signature | Digital Content | d |
|  |  |  |  |  |  |  |  | 20.9 | could download or print the signed document | Participants receive a copy of the completed consent form | Accessibility | a |
| 21 | (Ramos 2017) | User-centered Design, Experience, and Usability of an e-Consent User Interface to Facilitate Informed Decision Making in an HIV Clinic | North America | Original Research | Randomised Controlled Trial | Patients with HIV | 5 | 21.1 | visual animations and icons that convey important elements | Support interactive digital components such as online quiz, graphical media and audio-visual aid | Digital Content | d |
|  |  |  |  |  |  |  |  | 21.2 | simplified text headings designed to easily describe information | Support interactive digital components such as online quiz, graphical media and audio-visual aid | Digital Content | d |
|  |  |  |  |  |  |  |  | 21.3 | an electronically generated document as a PDF file | Participants receive a copy of the completed consent form | Accessibility | a |
|  |  |  |  |  |  |  |  | 21.4 | usability through navigation of highlighted icons and visible user functions, such as ‘home page’ and ‘next’ buttons | Establish tracking mechanism for separate consents | Accessibility | a |
| 22 | (Rau et al. 2020) | The generic Informed Consent Service gICS®: implementation and benefits of a modular consent software tool to master the challenge of electronic consent management in research | Europe | Review | Methodical/Framework Analysis | App Development | 8 | 22.1 | uses SignPads for obtaining and recording digital signatures with biometric values (e. g. pressure, speed and fluency of writing movement) | Use validated electronic signature | Digital Content | d |
|  |  |  |  |  |  |  |  | 22.2 | allow tracking changes over time, e. g. through amendments or new research modules | Establish tracking mechanism for separate consents | Accessibility | a |
|  |  |  |  |  |  |  | 9 | 22.3 | The participant is enabled to change his/her will at any time. A given consent can be updated as well as withdrawn without any restrictions using gICS. For a precise chronological documentation every change results in a new “latest” and versioned consent easily manageable with gICS | Allow participant to amend their consent | Autonomy | f |
|  |  |  |  |  |  |  |  | 22.4 | Digitising and managing consents in gICS includes the possibility to upload one or more files (PDF‑format, jpg), e. g. a scanned document of the participant’s paper‑based consent, to the digital consent | Establish tracking mechanism for separate consents | Accessibility | a |
| 23 | (Rothwell et al. 2014) | A Randomized Controlled Trial of an Electronic Informed Consent Process | North America | Original Research | Randomised Controlled Trial | Electronic Health Record | 4 | 23.1 | present an explanation in words and pictures than solely in words | Support interactive digital components such as online quiz, graphical media and audio-visual aid | Digital Content | d |
|  |  |  |  |  |  |  |  | 23.2 | present corresponding words and pictures contiguously rather than separately | Support interactive digital components such as online quiz, graphical media and audio-visual aid | Digital Content | d |
|  |  |  |  |  |  |  |  | 23.3 | Present words as auditory narration rather than as visual onscreen text | Support interactive digital components such as online quiz, graphical media and audio-visual aid | Digital Content | d |
|  |  |  |  |  |  |  |  | 23.4 | Use few rather than many extraneous words and pictures | Simplify the language | Language | l |
| 24 | (Schneiderheinze et al. 2019) | Development and Usability Analysis of a Multimedia eConsent Solution | Europe | Original Research | Randomised Controlled Trial | App Development | 299 | 24.1 | the content is presented by a speaker and illustrated in parallel by animated pictograms | Provide interactive personnel to optimize comprehension and trust | Comprehension Engagement | c |
|  |  |  |  |  |  |  |  | 24.2 | there are several buttons to interact with at the bottom of the screen | Support interactive digital components such as online quiz, graphical media and audio-visual aid | Digital Content | d |
|  |  |  |  |  |  |  |  | 24.3 | PDF button displays the complete patient information in PDF format | Participants receive a copy of the completed consent form | Accessibility | a |
| 25 | (Simon et al. 2014) | Traditional and Electronic Informed Consent for Biobanking: A Survey of U.S. Biobanks | North America | Original Research | Interviews/Surveys | Electronic Health Record | 427 | 25.1 | Access to good technical support and an efficient backup plan | Support information technology infrastructure | Digital Content | d |
|  |  |  |  |  |  |  |  | 25.2 | linking of clinical and research consents to a multi-institutional health data collection system | Integrate research consent data with electronic health record | Accessibility | a |
|  |  |  |  |  |  |  |  | 25.3 | consent documents are presented in simple HTML text format or supported by graphics, video, voice narration, and/or other features | Support interactive digital components such as online quiz, graphical media and audio-visual aid | Digital Content | d |
|  |  |  |  |  |  |  |  | 25.4 | E-capture of signatures will allow complete electronic filing and storage of signed consent documents | Use validated electronic signature | Digital Content | d |
|  |  |  |  |  |  |  |  | 25.5 | authentication issues on signature | Use validated electronic signature | Digital Content | d |
| 26 | (Simon et al. 2018) | Perspectives on Electronic Informed Consent From Patients Underrepresented in Research in the United States: A Focus Group Study | North America | Original Research | Randomised Controlled Trial | Patients Underrepresented in Research | 3 | 26.1 | interactive, multiple-choice questions were inserted at relevant points in the slideshow to emphasize critical concepts | Provide interactive personnel to optimize comprehension and trust | Comprehension Engagement | c |
|  |  |  |  |  |  |  |  | 26.2 | could select options in the slideshow to go to the next slide, go to the previous slide, replay the narration, mute the narration, or change the volume of the narration | Participant is always able to control and restrict the access to the shared record | Autonomy | f |
|  |  |  |  |  |  |  |  | 26.3 | Each slideshow was accessed from a website and could be viewed using desktop computers, laptops, tablet computers, or smartphones | Integrate research consent data with electronic health record | Accessibility | a |
| 27 | (Stevens et al. 2016) | Risk based survey evidence supports electronic informed consent as a recruitment method for UK clinical trials | Europe | Perspective | Randomised Controlled Trial | Electronic Health Record | 134 | 27.1 | patient confidentiality, rights, and well-being; especially the ability of the researcher to assess capacity to consent | Allow participant to amend their consent | Autonomy | f |
|  |  |  |  |  |  |  |  | 27.2 | should be controlled and validated, with accurate, legible, contemporaneous, original, attributable, complete, and consistent data with a full audit trail | Fulfill general requirements and elements of informed consent | Comprehension Engagement | c |
|  |  |  |  |  |  |  |  | 27.3 | Robust security procedures must be in place | Provide secured and proper authentication | Confidentiality | y |
|  |  |  |  |  |  |  |  | 27.4 | possible to securely copy and print the data, storage, and archiving must allow reconstruction at a later date | Provide secured and proper authentication | Confidentiality | y |
|  |  |  |  |  |  |  |  | 27.5 | technology required to implement e-ICF is already well recognized and available | Support interactive digital components such as online quiz, graphical media and audio-visual aid | Digital Content | d |
| 28 | (Vanaken et al. 2019) | Awareness and Collaboration Across Stakeholder Groups Important for eConsent Achieving Value-Driven Adoption | Europe | Original Research | Interviews/Surveys | Stakeholder Groups | 733 | 28.1 | Ensure interface is user-friendly | Support interactive digital components such as online quiz, graphical media and audio-visual aid | Digital Content | d |
|  |  |  |  |  |  |  |  | 28.2 | Develop appropriate device management and helpdesk approach | Integrate research consent data with electronic health record | Accessibility | a |
|  |  |  |  |  |  |  |  | 28.3 | Design appropriate operational (eg, how to use metadata) and technical documentation and training, in line with site preferences | Integrate research consent data with electronic health record | Accessibility | a |
|  |  |  |  |  |  |  |  | 28.4 | Execute support activities including helpdesk and troubleshooting manuals | Integrate research consent data with electronic health record | Accessibility | a |
|  |  |  |  |  |  |  |  | 28.5 | Ensure interface is user-friendly and fit for participants | Support interactive digital components such as online quiz, graphical media and audio-visual aid | Digital Content | d |
|  |  |  |  |  |  |  |  | 28.6 | Conduct focus groups to receive feedback on eConsent tools | Support social annotation whereby participants can see each other comments for discussions | Comprehension Engagement | c |
|  |  |  |  |  |  |  |  | 28.7 | Ensure consent language is transparent about participant data collection | Simplify the language | Language | l |
|  |  |  |  |  |  |  |  | 28.8 | Develop training in line with participant population | Support social annotation whereby participants can see each other comments for discussions | Comprehension Engagement | c |
|  |  |  |  |  |  |  |  | 28.9 | Communicate to understand if eConsent design is compliant with local regulations | Support social annotation whereby participants can see each other comments for discussions | Comprehension Engagement | c |
|  |  |  |  |  |  |  |  | 28.10 | Consider using workarounds if the collection of participant data is not immediately achievable | Integrate research consent data with electronic health record | Accessibility | a |
|  |  |  |  |  |  |  |  | 28.11 | Collaborate with Ethics Committees to develop review process in line with technological and operational needs | Allow IRB oversight and amendment | Accessibility | a |
| 29 | (Varnhagen et al. 2005) | How Informed Is Online Informed Consent? | North America | Original Research | Methodical/Framework Analysis | Electronic Health Record | 42 | 29.1 | read the informed consent documents online in one of the two formats (paper or online-based) | Undertaking the consent process before the day intervention | Comprehension Engagement | c |
|  |  |  |  |  |  |  |  | 29.2 | click the “I accept” button should they wish to participate | Participant is always able to control and restrict the access to the shared record | Autonomy | f |
|  |  |  |  |  |  |  |  | 29.3 | Time to read the document was recorded on the computer and measured from the participant’s click to access the informed consent document to the click to consent to participate | Participant is always able to control their own pace during consent process | Comprehension Engagement | c |
| 30 | (Vayena et al. 2013) | Caught in the Web: Informed Consent for Online Health Research | Europe | Perspective | Methodical/Framework Analysis | Electronic Health Record | 2 | 30.1 | information actively supplied by the user (medical histories, genomic data, and Web posts) | Integrate research consent data with electronic health record | Accessibility | a |
|  |  |  |  |  |  |  |  | 30.2 | personal information collected by the Web site while the user is interacting with the site (IP and e-mail addresses, searches, and location data) | Integrate research consent data with electronic health record | Accessibility | a |
|  |  |  |  |  |  |  |  | 30.3 | users understand or are aware of the potential uses of their data when they access a site | Undertaking the consent process before the day intervention | Comprehension Engagement | c |
| 31 | (Weber et al. 2020) | A FHIR-Based eConsent App for the Digital Hospital | Europe | Review | Scoping Review | App Development | 4 | 31.1 | allow patients to consent and withdraw at any time | Allow participant to amend their consent | Autonomy | f |
|  |  |  |  |  |  |  |  | 31.2 | define the terms and conditions for projects using their data and samples (dynamic consent) | Use only explicit consent | Autonomy | f |
|  |  |  |  |  |  |  |  | 31.3 | help patients investigate projects their data and samples are being used | Establish digital technology for participants to track the use of their biological sample over time | Digital Content | d |
|  |  |  |  |  |  |  | 5 | 31.4 | patients signed on screen with a stylus pen | Use validated electronic signature | Digital Content | d |
|  |  |  |  |  |  |  |  | 31.5 | it provides sections for introductions, study information, privacy, usage of collected data and information on withdrawal of consent | Allow participant to amend their consent | Autonomy | f |
|  |  |  |  |  |  |  |  | 31.6 | Each section is displayed on screen for the patient to read and scroll through | Support interactive digital components such as online quiz, graphical media and audio-visual aid | Digital Content | d |
|  |  |  |  |  |  |  |  | 31.7 | Every section can show a” learn more” link , which navigates the patient to a different screen for more information | Establish tracking mechanism for separate consents | Accessibility | a |
|  |  |  |  |  |  |  |  | 31.8 | To start the consent process, a custom label reader was implemented to read a standard patient bar code and retrieve patient data needed for the consent document, such as name, date of birth and patient ID | Integrate research consent data with electronic health record | Accessibility | a |
| 32 | (Wilbanks 2020) | Electronic Informed Consent in Mobile Applications Research | North America | Perspective | Methodical/Framework Analysis | Electronic Health Record | 147 | 32.1 | participant has the right to opt out of the electronic process and request a paper-based consent form | Allow participant to amend their consent | Autonomy | f |
|  |  |  |  |  |  |  |  | 32.2 | must state that “significant new findings developed during the course of the research that may affect the subject’s willingness to continue participation” be provided electronically | Participant is always able to control and restrict the access to the shared record | Autonomy | f |
| 33 | United States Food and Drug Administration (FDA), 2015 (https://www.fda.gov/downloads/Drugs/GuidanceComplianceRegulatoryInformation/Guidances/UCM436811.pdf?source=govdelivery&utm_medium=email&utm_source=govdelivery) | Use of Electronic Informed Consent in Clinical Investigations Questions and Answers Guidance for Industry Draft Guidance [Internet] | North America | Perspective | Methodical/Framework Analysis | Regulations and Guidelines | 4 | 33.1 | eIC should be easy to navigate, allowing the user to proceed forward or backward within the system and to stop and continue at a later time | Participant is always able to control their own pace during consent process | Accessibility | a |
|  |  |  |  |  |  |  |  | 33.2 | Hyperlinks may be provided where helpful | Support interactive digital components such as online quiz, graphical media and audio-visual aid | Digital Content | d |
|  |  |  |  |  |  |  |  | 33.3 | eIC may also incorporate electronic strategies to encourage subjects to access all of the consent material before documenting their consent | Undertaking the consent process before the day of intervention | Comprehension Engagement | c |
|  |  |  |  |  |  |  |  | 33.4 | subjects should have the option to use paper-based or electronic informed consent methods completely or partially throughout the informed consent process | Allow participant to amend their consent | Autonomy | f |
|  |  |  |  |  |  |  | 5 | 33.5 | establish in-person discussions with study personnel or through a combination of electronic messaging, telephone calls, video conferencing, or a live chat with a remotely located investigator or study personnel. | Provide interactive personnel to optimize comprehension and trust | Comprehension Engagement | c |
|  |  |  |  |  |  |  |  | 33.6 | eIC may contain various methods to help an investigator assess the subject’s understanding of the information being presented during the eIC process | Support social annotation whereby participants can see each other comments for discussions | Comprehension Engagement | c |
|  |  |  |  |  |  |  |  | 33.7 | eIC may use interactive electronic-based technology, which may include diagrams, images, graphics, videos, and narration | Support interactive digital components such as online quiz, graphical media and audio-visual aid | Digital Content | d |
|  |  |  |  |  |  |  |  | 33.8 | eIC should be appropriate for the intended audience, taking into consideration the subject’s age, language, and comprehension level. | Identify the demographics and needs of the participants | Accessibility | a |
|  |  |  |  |  |  |  |  | 33.9 | eIC may include an electronic method to capture the signature of the subject or the subject’s LAR | Use validated electronic signature | Digital Content | d |
| 34 | Lawrence et al., 2017 (https://trialinnovationnetwork.org/wp-content/uploads/2017/12/2017-12-14-11.00-STRIDE-_-eConsent_-Moving-Towards-Personalized-Informed-Consent.mp4) | STRIDE & eConsent: Moving Towards Personalized Informed Consent [Internet] | North America | Perspective | Methodical/Framework Analysis | Regulations and Guidelines | Online page | 34.1 | Patients can 'sign' an eConsent document by: i) typing in their name, ii) signing their name via stylus/finger, or iii) entering a personalized PIN number | Use validated electronic signature | Digital Content | d |
|  |  |  |  |  |  |  |  | 34.2 | eConsent platform will use Avatars to guide participants through the consent process | Support interactive digital components such as online quiz, graphical media and audio-visual aid | Digital Content | d |
|  |  |  |  |  |  |  |  | 34.3 | scripted voice-over | Simplify the language | Language | l |
|  |  |  |  |  |  |  |  | 34.4 | Avatar can respond to questions, repeat information and provide additional information | Provide interactive personnel to optimize comprehension and trust | Comprehension Engagement | c |
|  |  |  |  |  |  |  |  | 34.5 | Participants may hover-over keywords to see pronunciation, definitions or more information about a word (gives participants ownership) | Support interactive digital components such as online quiz, graphical media and audio-visual aid | Digital Content | d |
|  |  |  |  |  |  |  |  | 34.6 | Researcher will determine keywords and information that appears | Support interactive digital components such as online quiz, graphical media and audio-visual aid | Digital Content | d |
|  |  |  |  |  |  |  |  | 34.7 | Virtual repository to serve as an institution-wide 'lock box' for consents | Establish digital storing biometric information on cloud storage | Digital Content | d |
|  |  |  |  |  |  |  |  | 34.8 | specific meta-data elements can be used to link consents in the repository to their respective studies | Integrate research consent data with electronic health record | Accessibility | a |
